# Effectiveness of a cognitive behavioral intervention in patients with medically unexplained symptoms: cluster randomized trial

**DOI:** 10.1186/1471-2296-13-35

**Published:** 2012-05-02

**Authors:** Alberto López-García-Franco, Mª Isabel del-Cura-González, Luis Caballero-Martinez, Teresa Sanz-Cuesta, Marta Isabel Díaz-García, Mª Teresa Rodriguez-Monje, Marcela Chahua, Inmaculada Muñoz-Sanchez, Dolores Serrano-González, Teresa Rollán-Llanderas, Esther Nieto-Blanco, Liliana Losada-Cucco, Fernando Caballero-Martínez, Nuria Sanz-García, Belén Pose-García, Montserrat Jurado-Sueiro, Manuela Luque Rey, Francisca García de Blas González, Mª Angeles Miguel Abanto, Teresa Sanz Bayona, Rafaela Ayllón-Camargo, Inmaculada Santamaría Lopez, María Luisa Santiago Hernando, Rosario Beltran-Alvarez, Ana Isabel Aguilar-Gutierrez, Jose Luis Mota-Rodriguez, Rafaél Cosculluela-Pueyo, Teresa López-Martín-Aragón, Rosa Bonilla-Sanchez, Mª Carmen Aritieda-González-Granda, Raquel Razola-Rincón, Mª Angeles Sanchez-de-la-Ventana, Concepción Martinez-Guinea, Luis Huerta-Galindo, Ana Belén Barrio-Ovalle, Susana Miguel-Martín, Paz Portero-Fraile, Higinio Pensado-Freire, Mª Luisa Herrera-Garcia, Amaya Azcoaga-Lorenzo, Inés Gómez-García, Nuria Llamas-Sandino, Isabel López-Borja, Hortensia Maldonado-Castro, Patricia Lumbreras-Villarán, Carlos Ascanio-Durán

**Affiliations:** 1 Centro de Salud Dr Mendiguchia, Leganés, Madrid, Spain; 2 Unidad de Apoyo a la investigación, Gerencia de Atención Primaria, Madrid, Spain; 3 Facultad de Ciencias de la Salud, Universidad Rey Juan Carlos, Alcorcón, Madrid, Spain; 4 Hospital Puerta de Hierro, Majadahonda, Madrid, Spain; 5 Facultad de psicología, Universidad de Educación a Distancia, Madrid, Spain; 6 Centro de Salud M, Ángeles López Gómez, Leganés, Madrid, Spain; 7 Escuela Nacional de Sanidad, Instituto de Salud Carlos III, Madrid, Spain; 8 Centro de Salud Huerta de los Frailes, Leganés, Madrid, Spain; 9 Centro de Salud Maria Jesús Hereza, Leganés, Madrid, Spain; 10 Centro de Salud Argüelles, Madrid, Spain; 11 Facultad de ciencias biosanitarias, Universidad Francisco de Vitoria, Pozuelo de Alarcón, Madrid, Spain; 12 Centro de Salud Castilla la Nueva, Fuenlabrada, Madrid, Spain; 13 Centro de Salud Cuzco, Fuenlabrada, Madrid, Spain; 14 Centro de Salud Boadilla, Boadilla del Monte, Madrid, Spain; 15 Centro de Salud Pozuelo Estación, Pozuelo de Alarcón, Madrid, Spain; 16 Centro de Salud Sierra de Guadarrama, Collado Villalba, Madrid, Spain; 17 Centro de Salud Francia, Fuenlabrada, Madrid, Spain; 18 Centro de Salud Loranca, Fuenlabrada, Madrid, Spain; 19 Centro de Salud Valdezarza, Madrid, Spain

## Abstract

**Background:**

Medically unexplained symptoms are an important mental health problem in primary care and generate a high cost in health services.

Cognitive behavioral therapy and psychodynamic therapy have proven effective in these patients. However, there are few studies on the effectiveness of psychosocial interventions by primary health care. The project aims to determine whether a cognitive-behavioral group intervention in patients with medically unexplained symptoms, is more effective than routine clinical practice to improve the quality of life measured by the SF-12 questionary at 12 month.

**Methods/design:**

This study involves a community based cluster randomized trial in primary healthcare centres in Madrid (Spain). The number of patients required is 242 (121 in each arm), all between 18 and 65 of age with medically unexplained symptoms that had seeked medical attention in primary care at least 10 times during the previous year. The main outcome variable is the quality of life measured by the SF-12 questionnaire on Mental Healthcare. Secondary outcome variables include number of consultations, number of drug (prescriptions) and number of days of sick leave together with other prognosis and descriptive variables. Main effectiveness will be analyzed by comparing the percentage of patients that improve at least 4 points on the SF-12 questionnaire between intervention and control groups at 12 months. All statistical tests will be performed with intention to treat. Logistic regression with random effects will be used to adjust for prognostic factors. Confounding factors or factors that might alter the effect recorded will be taken into account in this analysis.

**Discussion:**

This study aims to provide more insight to address medically unexplained symptoms, highly prevalent in primary care, from a quantitative methodology. It involves intervention group conducted by previously trained nursing staff to diminish the progression to the chronicity of the symptoms, improve quality of life, and reduce frequency of medical consultations.

**Trial registration:**

The trial was registered with ClinicalTrials.gov, number NCT01484223 [ http://ClinicalTrials.gov].

## **Background**

### **The somatoform disorders**

Somatic symptoms are those symptoms that cannot be medically explained and when they only have a somatic nature, they are referred to as somatoform disorders. They can be found in a healthy population as well as in patients with psychiatric disorders; and they represent an important reason for doctor’s examination in primary care. This group of disorders includes [1]: disorder caused by somatization (including 8 symptoms), syndrome by abbreviated somatization (4 symptoms throughout the whole life in men or 6 in women), or multi-somatoform disorder (three or more important symptoms without medical explanation and more than 2 years of development).

The diagnosis of these disorders is difficult. The classification of somatoform disorders in CIE-10 and in DSM IV is shown in Table 1.

**Table 1:** Somatoform disorders classification

	**International Classification of Diseases, 10th version (ICD-10)**		**Diagnostic and Statistical Manual of Mental Disorders, 4th Edition (DSM-IV)**
**F45**	**Somatoform disorders**		**Somatoform disorders**
F45.0	Somatization disorder	300.81	Somatization disorder
F45.1	Undifferentiated somatoform disorder	300.82	Undifferentiated somatoform disorder
F45.2	Hypochondriacal disorders	300.7	Hypochondriasis
F45.3	Somatoform autonomic dysfunction	300.7	Body Dysmorphic Disorder
.30	Heart and cardiovascular system		
.31	Upper gastrointestinal tract	307.8	Pain Disorder Associated with Psychological Features
.32	Lower gastrointestinal tract	300.11	Conversion Disorder
.34	Genitourinary system		
.38	Other organ or system		
F45.4	Persistent somatoform pain disorder		
F45.8	Other somatoform disorders		
F45.9	Somatoform disorder, unspecified		

Today, there is debate about the suitability of somatization disorder differ from other functional symptoms. It is estimated that 75 % of patients with medically unexplained symptoms (MUS) and over-the health system does not meet the diagnostic criteria for abbreviated somatization syndrome [2]. While somatization disorder affecting 0.4 % of the population, all somatoform disorders are between 20 and 30 % of consultations in primary care [2]. When the patient has at least one symptom in 5 specific areas (pain, fatigue, irritable bowel syndrome, somatic symptoms of anxiety or somatic symptoms of depression) and protracted (at least 4 consultations in a year) some authors recommend defining it as MUS [3]

In a clinical series in the USA, a clear organic cause was not found among the 20 % - 84 % of the patients that went to the family physician with throbs, thoracic pain, migraine, fatigue or sickness [4]. On the other hand, up to 75 % of the patients with major depressive disorders or anxiety attacks go to their family doctors only because of the somatic symptoms they show [5].

A study in Spain [6] shows that 34.5 % of patients who visit for the first time to their family physician suffer from for somatic symptoms. Most of these symptoms are severe, and a percentage end up having a somatization disorder or undifferentiated somatization disorder.

The somatoform disorders are an excluding diagnosis that forces to carry out a significant number of complementary tests and derivations to the second and third level.

The somatoform disorders are highly prevalent in primary care [2]. It is estimated that this kind of patient uses up 14 times more resources in outpatient, and 6 times more expenses under hospital regimen [7].

### **Approach to the somatoform disorders**

There is consensus that the approach to the different features of somatoform disorders should be carried out with similar interventions; without indicating specific therapies for each of the aforementioned manifestations [8].

There is data on the usefulness of the implementation of Good Clinical Practice (GCP) criteria (Table 2), by the family physician; showing that they can relieve the symptoms, reduce the demand on attention, improve the patients’ satisfaction with the given attention and limit the costs; though with modest results [8]. It is important to find tools in the psychosocial approach of these patients added to those already recommended for GCP. The Cognitive Behavioral Therapy (CBT) or the psycho-dynamic psychotherapy has been useful in the field of secondary medical care [9-11]. In a recent systematic review of the 34 analyzed RCTs on interventions in somatoform symptoms, only 9 are developed in primary care [12]. In these nine RCTs conclude significant results in the decrease of use of services [13-16], improvement on physical and/or psychical performance [17-20]. The reattribution technique specifically designed by Goldberg and Cols [21] is used in two of the analyzed studies for the treatment of somatization in primary care through the teaching and training of family physicians. Their results show low improvement on the physical symptoms [22,23] but there is a significant decrease in the prescription of analgesic medication (8.1 %-23 %) and anti-depressants (11.3 %-26.9 %). The interventions carried out by nursing professionals are effective, but they are excessively expensive in terms of number of sessions (at least 12) and in the required training time (80 hours) [8].

**Table 2:** Regulations on good clinical practice in the general approach of the patient with medical unexplained symptoms

	
1. To plan regular appointments every 4–6 weeks in order to treat them clinically during the first year / 6 months, or if a new symptom comes up (in worsening periods, appointments could be more frequent).	
2. To give the patient a detailed document on the origin of the symptoms	
3. To establish high-priority objectives	
4. To restrict complementary examinations to the most indispensable ones	
5. To control the visits to specialists	
6. To have the patient treated by only one doctor	
7. To calm down and to reassure	
8. To identify the psychosocial stimuli that are involved as well as their link to the worsening of the symptomatology	
9. To avoid ambiguous information about the findings that come up	
10. To avoid spurious diagnostics	
11. Not to treat what the patients do not suffer from	
12. To avoid dichotomy explanations, i.e. (mental-physical nature)	
13. To mediate, when possible, in their psychosocial problems	
14. The best policy is to be sincere on the reports	
15. To approach some problems in a multidisciplinary way	
16. To organize the management/treatment of the difficult cases	
17. To be consistent with the approaches	
18. To properly remit to the psychiatry services	

An alternative for the treatment of this kind of disorder in the field of primary care could be group interventions with a limited number of sessions. Martin A et al [24] carry out an RCT that assessed the effectiveness of a single cognitive-behavioral group session (relaxation and motivation technique to take physical exercise) in order to improve the approach of the patient to the symptoms, by means of their explanation from a non- biologist focus. They found a significant reduction in the seriousness of the symptoms, in the number of consultation to the family physicians (not in the visits to the specialist), reduction in number of drug (prescriptions) and in the number of days on sickness absence. This intervention was carried out by mental health professionals, which limits its external validity and its implementation in the field of primary care.

A correct approach to these patients by their family doctor may reduce iatrogenic. The training of primary care professionals can help to improve care for patients consulting for MUS.

A model on cognitive-behavioral group intervention, based on reattribution techniques, carried out through four group sessions by primary care nurses, together with the implementation of regulations on GCP, would be effective for improving quality of life of patients with MUS.

### **Aims**

The aim of the present work is to determine whether a cognitive-behavioral intervention group in patients between 18 and 65 years old with medically unexplained symptoms, is more effective than routine clinical practice to improve the quality of life measured by SF-12 questionnaire at one year follow-up.

The secondary objectives are to determine whether a cognitive-behavioral intervention group with medically unexplained symptoms, is more effective than routine clinical practice to:

– Improve the quality of life measured by SF-12 questionnaire at 3 and 6 months.

– Reduce the drug consumption.

– Reduce the number of medical consultation.

– Reduce the days of sickness absence.

– Raise the perception on improvement (standardized global vision) from the patient's point of view (PGI-I questionnaire) and from the doctor’s (with the CGI-I).

## **Methods/Design**

### **Design of the study**

This study is a community, parallel clinical trial, randomised by clusters, that compares cognitive-behavioral intervention group with routine clinical practice. The intervention will be carried out by health professionals in 14 Primary Health Care Centres (PHCCs), in Madrid (Spain).

The randomization units will be the PHCCs (clusters). The units of analysis will be the patients between 18 and 65 years old with MUS who have seeked medical attention in PHCCs at least 10 times over the past year. The design by clusters minimised possible contamination effects between centres.

### **Subjects of the study**

Adult patients between 18 and 65 years old with MUS, defined by the occurrence of two or more symptoms during the last 6 months without any evidence of organic disease that could explain them, and who have seeked medical attention in primary care at least 10 times during the last year.

1) *Inclusion criteria*. All patients were required to be:

a. Be able to meet the demands inherent to the trial, i.e, to have no intention of moving, to be reachable for the next year, and to have the capacity to understand the questionnaires presented

b. Give signed informed consent to be included in the study, and to meet no exclusion criterion.

2) *Exclusion criteria.*

a. Diagnosis of severe mental disorder.

b. Suicidal ideation at the time involved in the study.

c. Diagnosis of addiction to toxic substances.

d. Diagnosis of organic disease responsible for the symptoms.

e. Previous psychotherapy during the previous year.

#### ***Randomisation***

The randomization unit will be the PHCC. An independent statistician will randomly assign the 14 PHCCs (7 centres in each group) to either the intervention group or a control group following a simple, computer-generated random sequence (Epidat 3.1 software).

Consecutive patients will be chosen to minimize the risk of bias in their selection.

During consultations, patients will be informed about the study and asked whether they would like to take part. Those who accept will be asked to give their signed consent, and checks were made to ensure they met all inclusion criteria but no exclusion criteria.

#### ***Sample size***

##### *Method of calculation*

For an alpha of 0.05, a power of 80 %, and in order to detect an increase of 20 % in the proportion of patients that improve at least 4 points on area of mental healthcare of the SF-12 questionnaire (50 % in the intervention group and 30 % in the control group), the overall sample size required is 206 patients (103 in each arm of the study).

Since randomization is by clusters, the sample size had to be larger than if simple randomisation had been performed, in order to take into account the design effect. The intra-class correlation coefficient is 0.01 [25,26] and a mean cluster size is assumed to be 8 patients. The design effect is 1.07. Given these assumptions, and expecting a 10 % loss rate at one year, the final sample size required is 242 (121 in each arm).

### **Masking**

In a study of this type it is impossible to mask the intervention. The analysis data will be performed by independent professionals blinded to the assignment group.

Intervention:

a. In the control group:

Routine clinical practice: The clinical approach of the patients will be done following the recommendations of clinical practice guidelines as detailed in Table 2.

b. In the intervention group:

Besides the routine clinical practice, four weekly cognitive-behavioral group sessions of 2 hours guided by nurse professionals will be carried out in the PHCC. The groups will be made up by 6–12 patients.

The content of the sessions will be as follows (Table 3):

**Table 3:** Structure of the group sessions in the intervention group


1.	COGNITIVE REATTRIBUTION
	· Psychological explanation of the symptoms: To explain the objective of the session that is not precisely to heal the symptoms, but rather “to improve doping". To explain that somatization is a common phenomenon resulting from different factors and mechanisms. The patients are asked to talk about the different factors; and stress is introduced as an explanatory element of the symptoms.
	· The importance of the cognitive issue. They give examples on the role of knowledge and the patients are asked to link them to their own experiences. The objective is to show how the behaviors and the attitude toward the symptoms could change the response to them.
2.	RELAXATION AND SYMPTOMATIC RELIEF
	· Once the role of stress in the genesis of pain has been described, techniques are introduced where physical exercising and muscular relaxation are combined. The patients are provided with a CD with spoken instructions.
3.	BEHAVIOR-ILLNESS
	· The patients are given details on optional treatments and on the correct use of sanitary services. They are also instructed on the mechanisms to control anomalous behavior toward the illness.
4.	AEROBIC EXERCISING
	· To encourage physical exercising: to do away with the bad habit of stating that “pain stops me from doing any kind activity” thus, they avoid doing any kind of activity. An ongoing program on physical exercising is outlined.

If the study results prove that the intervention is effective, cognitive behavioral sessions are planned for the patients in the control group.

Before starting the intervention itself, will develop the training for professionals involved in the study. In order to make the best use of the diagnosis and the therapeutic approach on the patients that have been identified as suffering from anxiety and/or depression disorders, and to standardize clinical practice, a two-hour initial training session will be carried out for all the doctors that participate in the study. The session will have the following content: train in the use of the PRIME-MD questionnaire; as well as in upgrading the rules on pharmacological treatment of the detected pathologies. In addition will be reviewed Standards for GCP for approaching the patients with MUS (Table 2).

Besides, there will be a ten-hour specialized training session aimed to the nurses in charge of perform the intervention at each center, reattribution techniques and cognitive-behavioral, that underlie the intervention group.

### **Data collection method**

The doctor will recruit the patients and will assess the selection criteria. He will let the patient know about the objectives of the study; and will request his informed consent.

All patients who accepted to participate will get the SF-12 questionnaire on quality of life at the end of the doctor’s examination. Furthermore, all patients will get the PRIME-MD questionnaire to identify any psychiatric co-morbidity that will be treated by the doctor according to his criterion.

At 3, 6 and 12 months (visits V2, V3 and V4, respectively) will be referred the patient to evaluate the usefulness of cognitive-behavioral sessions, and completion of quality of life questionnaire (SF-12) and Clinical Global Impression of patient and physician (PGI and CGI).

The structure of the study is described in Table 4.

**Table 4:** Project structure

	**Intervention Group**	**Control Group**
**Recruitment visit (V 0)**	Selection criteria	
Consecutive selection	Request on informed consent	
	PRIME MD Questionnaire	
	Variables:	
	· SF-12 Questionnaire on life quality	
	· Descriptive variables: sex, cultural level, age, months with symptoms, time on antidepressants consumption, requested tests y specialized derivations to	
**Training of professionals involved**	Training in management of PRIME-MD survey. Review of Standards of Good Clinical Practice	
	Training in cognitive-behavioral (Only intervention Group)	
**Baseline visit (V1)**	Variables:	
	· SF-12 Questionnaire	
	· Prescribed medicine	
	· Requested tests	
	· Derivations to secondary level	
	· Days of sickness absence	
	· Assessment of compliance with the standards of good clinical practice	
	· Questionnaire on global clinical impression; both, the doctor and the patient	
**Intervention**	There will be a weekly group session for 4 weeks (Only intervention Group)	
	Routine clinical practice	
**3-months post Intervention visit (V2)**	Variables:	
	· SF-12 Questionnaire	
	· Prescribed medicine	
	· Requested tests	
	· Derivations to secondary level	
	· Days of sickness absence	
	· Assessment of compliance with the standards of good clinical practice	
	· Questionnaire on global clinical impression; both, the doctor and the patient	
	· Assessment by the nurse subject participation.	
	· Assessment of cognitive- behavioral module by subject (Only intervention Group)	
**6-months post Intervention visit (V3) 12-months post Intervention visit (V4)**	Variables:	
	· SF-12 Questionnaire	
	· Prescribed medicine	
	· Requested tests	
	· Derivations to secondary level	
	· Days of sickness absence	
	· Assessment of compliance with the standards of good clinical practice	
	· Questionnaire on global clinical impression; both, the doctor and the patient	
	· Assessment of cognitive- behavioral module by subject (Only intervention Group)	

### **Variables**

#### ***Outcomes variables***

The main outcome variable is the improvement in 4 or more points in the area of Mental Healthcare from the SF-12 Quality of life at 12 months. The Spanish version of this Questionnaire has been validated: high inner consistency (range 0.77-0.92) for all the dimensions, except social function (0.55). The questionnaire is self-administered.

The secondary outcome variables recorded are:

– Questionnaire on Global Clinical Impression. Used to assess the patient's (PGI questionnaire) and the doctor’s (CGI Questionnaire) perceptions on the its improvement with the interventions. Will be administered at 6 months (to assess the effectiveness of the rules of good clinical practice) and at 12 months to evaluate the effectiveness of the intervention group added to the standards of good clinical practice. Will be treated as ordinal qualitative variable.

– Quality of life at 3 and 6 months after the intervention measured by the SF-12 questionnaire.

– Number of consultations during the period of study (broken down between nursing and medicine, each group session will be counted as a nursing consultation).

– Prescribed medicine during the period of study, with doubtful therapeutic value for their processing; related to the MUS. The included medicines are those related to the functional symptomatology:

– Pain: analgesic medicines, Nonsteroidal Anti-inflammatory Drugs and muscular pain reliever.

– Dizziness: vestibular sedatives, peripheral brain and vasodilators.

– Digestive Symptoms: pro-kinetics, anti-emetics.

– Asthenia: vitamin complex, restoratives, derived from ginseng, appetite stimulants.

– Anxiety: anxiolytics, antidepressants.

It will be regarded as quantitative variable, defined as the number of medicine taken during the period of study.

– Days of sickness absence during the period of study.

#### ***Clinical Variables***

– Duration of the somatic symptoms before the study. Quantitative variable (number of months).

– Time on anti-depressants consumption (months) before the study.

– Psychiatric Co-morbidity at the beginning of the study. It will be analyzed as a dichotomy qualitative variable (yes/no); assessing the presence/absence of depression, anxiety and somatic-morph disorders (according to the PRIME MD classification).

– PRIME-MD questionnaire; validated in Spain [27] that categorizes the patient into 5 areas of mental disorders with greater incidence on primary care: disorders in the state of mind/mood, anxiety disorders, disorders on nutritional behavior, somatoform disorders and disorders due to alcohol abuse or addiction. There are two components: a questionnaire for the patient that acts as a sifting/testing tool, and an assessment guide for the doctor that allows the diagnostic confirmation on the case. Once the questionnaire has been carried out, the patients diagnosed under anxiety or depression will be treated in a standardized way. Thus a bias is eliminated in the understanding of the study results, i.e. not to have treated the identified anxious-depressive psychiatric co-morbidity in a proper way; an issue that could have positive effect on the somatoform manifestations.

– Derivations carried out at secondary level.

– Complementary Tests, grouped in the following categories: laboratory, image tests, and endoscopies.

#### ***Sociodemographics descriptive variables***

Age, sex, cultural level, labor status on date of inclusion into the study.

#### ***Withdrawal***

Subject withdrawal criteria

a. The appearance of a pathological condition to explain the symptoms.

b. The use of an unusual alternative therapy (other than those normally used by the patient) for the treatment of symptoms, such as psychotherapy, etc. that could alter the outcome.

c. If the patient shows his/her desire to withdrawing from the study.

d. If the patient does not come to the follow-up visits.

If any of these events take place, it will be registered on the Case Report Form (CRF); stating clearly the date and the reason. The data will be equally processed to allow the analysis with intention to treat.

### **Statistical Analysis**

The use of randomization by clusters conditions the statistical analysis that can be employed, especially in the calculation of confidence intervals and the testing of hypotheses. The following analyses will be undertaken:

1. **Descriptive analysis** of each variable with its corresponding CI at 95 %. Description of the profile of patients who abandoned the study plus their reason for withdrawal.

2. **Baseline comparison** of the two intervention groups in terms of the variables measured, prognostic factors and descriptive variables. Bivariate statistical tests will be used depending on the type of variable (qualitative or quantitative one).

3. **Analysis of primary outcome**: comparison of the proportion of patients that improve in 4 or more points in the area of Mental Healthcare from the SF-12 in both groups, at 12 months after patient inclusion, using the Chi-squared test, and the calculation of confidence intervals 95 %. Logistic regression with random effects will be used to adjust for prognostic factors; the dependent variable will be the improvement or not in 4 or more points obtained in Mental Healthcare; and the independent variable the intervention group to which each patient belonged. Confounding factors or factors that might alter the effect recorded will be taken into account in this analysis.

4. **Analysis of secondary outcomes variables**. The results of the secondary variables according to the assigned group will be compared for each secondary response variable using appropriate statistical tests.

All statistical tests will be performed with intention to treat. The last observation carried forward will be used for missing data. Significance was set at p < 0.05. All calculations will be made using SPSS© v.18 software.

### **Ethical considerations**

The study protocol was approved by the Research Ethics Committee on October, 29^th^ 2008 and met all good clinical practice demands right. This study has been funded by the Spanish Ministry of Science and Innovation via the Instituto de Salud Carlos III (PI08/90707). The trial was registered with ClinicalTrials.gov, number NCT01484223 [ http://ClinicalTrials.gov].

## **Discussion**

Resolute treatment of psychological or psychosocial issues by primary care professionals is a encouraging challenge. The study incorporates the PRIME MD questionnaire, a useful tool for diagnosing comorbid conditions. The time for application is 10 minutes. Its sensitivity and specificity make it a valid tool for the diagnosis of the most prevalent psychiatric disorders in primary care.

The study design used in this work is, however, subject to certain limitations. The intervention cannot be masked, which could influence the assessment of its effect. Certainly, the possibility of contamination exists if the patients or professionals involved communicate with one another. For this reason randomization by clusters was employed. It was assumed that the number of clusters was sufficient for the random assignment of PHCCs to one arm or the other to compensate for such potential confounding factors.

To mask the analysis of data, the persons charged with this task were blind to the arm to which each health care center had been assigned.

## **Abbreviations**

CBT: Cognitive behavioral therapy; CGI: Clinical global impression; ICD: International statistical classification of diseases and related health problems; CRF: Case report form; DSM-IV: Diagnostic and statistical manual, mental disorders-IV; GCP: Good clinical practice; PGI: Patient global impression; PHCC: Primary healthcare centre; PRIME-MD: Primary care evaluation of mental disorders; RCT: Randomized controlled trial; SF-12: Short-form health status measures-shorter.

## **Competing interests**

This manuscript contains no information on medical devices. The authors received, nor will receive, individual financing of their work.

## **Authors' contributions**

ALGF conceived of the study, participated in its design and coordination of the study. IDC, TSC, LC, and MIDG (senior authors) participated in the design of the study. ALGF, IDC, TSC, MTRM, PL and MC directed the writing of the manuscript. Contributions were made by the remaining authors. All authors read and approved the final manuscript.

## The SOMATIZADORES Group

Clinical assistance group:

**PHCC Mendiguchía Carriche (Leganés):** Alberto López García-Franco, Mar Álvarez Villalba, Nuria Sanz López, Belén Pose García, Amaya Azcoaga Lorenzo, Francisca García de Blas, Miguel Ángel Barajas.

**PHCC María Ángeles López Gómez “Pedroches” (Leganés):** Teresa Rodríguez Monje, Mercedes Fernández Girón, Liliana Losada Cucco, Jeanette Sánchez López , Beatriz López Serrano, María Cortés Durán. Dolores Serrano, Rafaela Ayllón Camargo, Imma Santamaría López.

**PHCC Cuzco (Fuenlabrada):** Mª Ángeles Miguel Abanto. Teresa Sanz Bayona, Teresa López Martin-Aragón.

**PHCC Castilla la Nueva (Fuenlabrada):** María Santiago Hernando, Carlos Díaz Gómez Calcerrada, Guadalupe García Caro. Monserrat Jurado Sueiro, Manuela Luque Rey.

**PHCC Boadilla del Monte (Boadilla del Monte):** Mª Fe Prádana Martínez, Lourdes Del Santo Mora, Ángeles García Pindado, Nuria Ruiz Hombrebueno. Rosario Beltrán Álvarez, Ana Isabel Aguilar Gutiérrez, José Luis Mota Rodríguez, Rafael Cosculluela Pueyo.

**PHCC Pozuelo Estación (Pozuelo de Alarcón):** José Mª Fernández-Bravo Álvarez, María Lacalle Rodríguez-Labajo, Diana Pitarch Velasco, Celia García López, Irene Ayuso Olmedo. Rosa Bonilla Sánchez, Mª Carmen Artieda-González-Granda, Raquel Razola Rincón.

**PHCC Sierra de Guadarrama (Collado Villalba):** Mª Gloria Heras Salvat. Mª Ángeles Sánchez de la Ventana, Concepción Martínez Guinea, Luis Huerta Galindo, Ana Belén Barrio Ovalle, Susana Miguel Martin, Paz Portero Fraile.

**PHCC Mª Jesús Hereza (Leganés):** Teresa Rollán Landeras, Mª Jesús Bedoya Frutos, Mª Angeles Rollán Hernández.

**PHCC Huerta de los Frailes (Leganés):** Inmaculada Muñoz Sánchez, Francisco Gutiérrez Ruiz, Susana Menéndez Álvarez.

**PHCC Francia (Fuenlabrada):** Julia Sánchez Miró. Higinio Pensado Freire (HP).

**PHCC Loranca (Fuenlabrada):** Arturo Rodríguez Cardoso, Mª José Rojas Giraldo. Marisa Herrera Garcia.

**PHCC Valdezarza (Madrid):** Eva Muro Díaz, Dolores Reguera de Castro, Paloma Rius Fortea. Inés Gómez García, Nuria Llamas Sandino, Isabel López Borja, Hortensia Maldonado Castro.

**PHCC Villanueva de la Cañada (Villanueva de la Cañada):** Cristina López Menéndez, Juana Blasco Albert.

**PHCC Pozuelo San Juan (Pozuelo de Alarcón):** Alicia Díaz Revilla, Fernando León Vázquez, Pilar Burón Martínez, Beatriz Fandiño García, Pedro Pablo Iglesias Dorado, Mª José Rey Álvarez.

## Pre-publication history

The pre-publication history for this paper can be accessed here:

http://www.biomedcentral.com/1471-2296/13/35/prepub
